# Radiological Classification and Management Algorithm of Petrous Apex Cholesterol Granuloma

**DOI:** 10.3390/jcm13092505

**Published:** 2024-04-24

**Authors:** Daniele Marchioni, Chiara Alberti, Nicola Bisi, Alessia Rubini

**Affiliations:** Department of Otorhinolaryngology-Head and Neck Surgery, Azienda Ospedaliero-Universitaria di Modena, Via del Pozzo 71, 41125 Modena, Italy; daniele.marchioni@unimore.it (D.M.); dr.ssa.alberti@gmail.com (C.A.); rubinialessia@gmail.com (A.R.)

**Keywords:** petrous apex, cholesterol granuloma, temporal bone, computed tomography, drainage, surgical excision

## Abstract

**Background**: Petrous apex cholesterol granulomas (PACGs) are benign inflammatory cystic lesions of the temporal bone. Usually, asymptomatic patients may develop symptoms as the lesions expand. The diagnosis is based on both CT and MRI scans and the management relies on “wait and scan” or surgery. This paper aims at evaluating surgical outcomes and proposing a CT-based classification and a management algorithm. **Methods**: Patients with PACGs who were surgically treated between 2014 and 2024 were included. Symptoms, imaging, approach type and complications were considered. CT scans were classified as Type A (preserved apex cellularity), Type B (erosion of the apex cellularity), and Type C (involvement of the noble structures bone boundaries). The possible connection of the lesion with the infracochlear, perilabyrinthine and sphenoidal cellularity was assessed. **Results**: Nineteen patients with symptoms like diplopia, headache and sensorineural hearing loss were included. According to our classification, 1/19 patients was Type A, 4/19 were Type B and 14/19 were Type C. Five patients underwent a total resection, seven a subtotal and seven a surgical drainage. Only two complications were recorded, and 17/19 patients showed symptom regression and stability during follow-up. **Conclusions**: While the management of PACGs is still controversial, according to our classification and surgical outcomes, Type A, being mostly asymptomatic, should be managed with “wait and scan”, Type B should undergo surgery when symptoms are present, while Type C should always undergo surgery because of their invasiveness and potential complications. When possible, a drainage should be attempted; otherwise, a surgical resection is chosen, and its completeness depends on the preoperative general and hearing status.

## 1. Introduction

Cholesterol granuloma is a benign inflammatory lesion that develops as a tissue reaction to blood degradation products. It appears as an intraosseous cyst filled with dark, sticky, chocolate brown fluid and granulation tissue, while microscopically, it is made up of many cholesterol crystals surrounded by multinucleated giant cells and granulation tissue with histiocytes, round cells, macrophages and many capillary-sized blood vessels [1,2].

The temporal bone is one of the most common locations for a cholesterol granuloma which most likely occurs in the petrous apex, approximately in 0.6 per 1 million individuals without gender specificity. It usually affects young or middle-aged patients, and it is frequently unilateral [3,4,5].

The etiology of cholesterol granuloma is still controversial. The most accepted theory is the one proposed by Jackler and Cho [6], which suggests that an exuberant pneumatization during adulthood creates bony defects in the marrow spaces between the mucosa-lined petrous apex cell tracts and the marrow. At this point, the coaptation of the richly vascularized marrow and mucosa leads to apical air cell hemorrhage. The bone-erosive behavior of the cholesterol granuloma of the petrous apex is due to the chronic blood seepage from this marrow fistula.

Patients may remain asymptomatic for long periods of time and the outbreak of symptoms is related to the expansion of the cyst. The most common symptom is hearing loss, which affects almost 50% of the patients, followed by headache, dizziness, facial palsy, facial pain or spasms and diplopia [4,7,8,9].

The gold standard diagnosis for the petrous apex cholesterol granuloma (PACG) is based on CT and MRI scans [2]. In case of involvement or erosion of the carotid artery canal or of a stenosis or a displacement of the internal carotid artery (ICA), a pre-operative study of the ICA itself through angiography, CT angiography or MRI angiography is mandatory [10,11,12].

Nowadays, there is no universally accepted radiological classification of PACGs, nor are there any guidelines in the literature about its management, yet two different strategies are proposed to handle this lesion: the “wait and scan” policy and a surgical treatment [13]. The “wait and scan” strategy is recommended for those asymptomatic patients or for symptomatic ones in poor general condition. These former patients represent the majority of the ones suffering from this disease and this choice is possible because of the benign nature and the slow growth of the cholesterol granuloma and also because spontaneous resolutions are described in the literature [8,13,14,15].

Surgery is chosen for those patients with symptoms or with a compression of the vital structures nearby, as shown by the scans [16,17]. Successful surgery in these cases leads to the resolution of symptoms and the avoidance of complications related to the state of the erosion (facial paralysis, hearing loss, meningitis). There are two main surgical options: the surgical drainage of the lesion or a complete surgical removal [17,18]. In the literature, several approaches to the lesion using lateral transtemporal corridors or anterior trans-sphenoidal corridors have been described. These approaches can either spare the hearing function or not. The approaches that preserve it are the infralabyrinthine, retrosigmoid, infracochlear, middle cranial fossa and trans-sphenoidal ones, while the ones that compromise the hearing function are the transcochlear and transotic approach, the translabyrinthine one and the previously mentioned ones combined with the infratemporal fossa Type B approach [19].

The aim of this study is analyzing the outcome of surgically treated patients with PACG and evaluating the radiological features of this lesion on a CT scan of the temporal bone in order to create a radiological classification that can be useful for the decision-making process.

## 2. Materials and Methods

A retrospective analysis was carried out from October 2014 to March 2024 at our center involving patients who had undergone skull base surgery. Out of approximately 1700 interventions performed on the skull base, only the patients who underwent surgery for symptomatic cholesterol granuloma of the petrous apex were included in the study.

Selection criteria:

Only the patients suffering from cholesterol granuloma of the petrous apex who underwent surgery at our department were included in our study.

Exclusion criteria:

Patients who underwent a surgical procedure for petrous apex lesions different from a cholesterol granuloma were excluded from our study, as well as patients with a cholesterol granuloma undergoing a “wait and scan” policy at our department.

In particular, in all cases we analyzed the following parameters: brain MRI signal features; temporal bone CT scan features; the hearing function of the patient, symptoms and the presence of cranial nerve deficits (III, IV, VI, VII, IX, X, XI, XII nerves).

### 2.1. MRI Radiological Evaluation

The signal of the lesion of the petrous apex was analyzed in T1-weighted sequences with and without contrast enhancement and in T2-weighted sequences, in order to carry out a correct diagnosis of the nature of the lesion.

### 2.2. CT Scan Imaging Study of the Petrous Apex

Once a PACG was diagnosed on the basis of the MRI signal, the CT scan of the petrous apex and the clivus were analyzed as well as the bone features of this anatomical region. Depending on the presence or absence of a petrous apex or clivus bone remodeling injury, we evaluated the following parameters: the presence of a preserved cellularity of the petrous apex, the degree of erosion and remodeling of the apex cellularity and the possible involvement of the bone limitations covering the noble structures such as the ICA, the internal auditory canal (IAC), the otic capsule, the dura mater and the jugular bulb.

**Figure 1:**
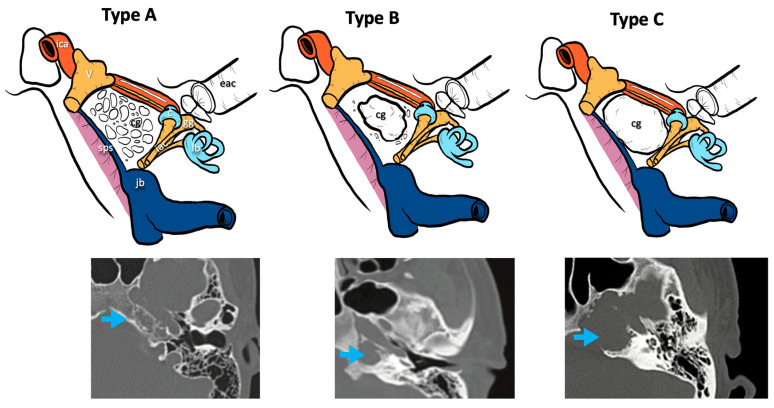
CT classification of cholesterol granulomas. V, fifth cranial nerve; ica, internal carotid artery; jb, jugular bulb; iac, internal auditory canal; lb, labyrinthine block; c, cochlea; eac, external auditory canal; gg, geniculate ganglion; sps, superior petrosal sinus; cg: cholesterol granuloma; blue arrows, cholesterol granuloma on the CT scans.

Three radiological types of cholesterol granuloma were distinguished according to the different radiological features of the petrous apex bone modifications and remodeling caused by this lesion (Figure 1):

- Type A cholesterol granuloma: presence of a cholesterol granuloma of the petrous apex with preserved cellularity in the absence of erosive phenomena and/or of cellular confluence.
- Type B cholesterol granuloma: presence of a cholesterol granuloma of the petrous apex with erosive phenomena and cellular confluence without any involvement of the bony limitations that cover the noble structures of the apex (otic capsule, IAC, middle and posterior cranial fossa dura, ICA, jugular bulb).
- Type C cholesterol granuloma: presence of a cholesterol granuloma of the petrous apex with erosive and cellular confluence phenomena with involvement of the bony limitations covering the noble structures of the apex (otic capsule, IAC, dura of the middle and posterior cranial fossa, ICA, jugular bulb).

Thanks to the CT scan, the anatomical condition of the petrous apex, in relation to the surrounding structures, was assessed as well as the presence of a connection between the cholesterol granuloma cyst and the infracochlear, perilabyrinthine and sphenoidal cellularity.

**Figure 2:**
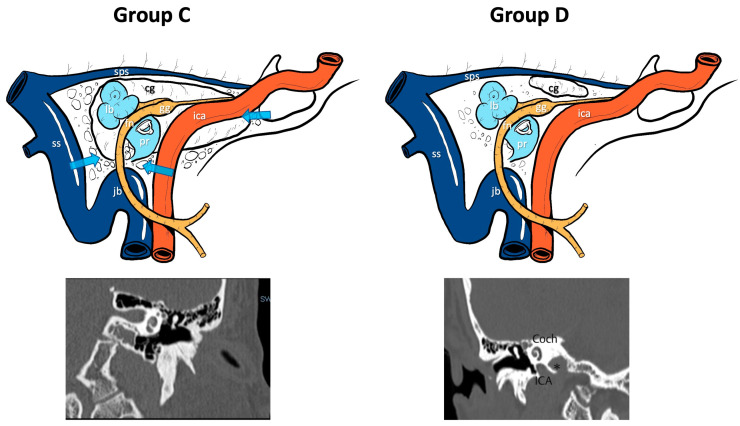
CT classification of the relationship between the cholesterol granuloma and the infracochlear, perilabyrinthine and sphenoidal air cells: ss, sigmoid sinus; sps, superior petrosal sinus; lb, labyrinthine block; fn, facial nerve; gg, geniculate ganglion; ica (or ICA), internal carotid artery; pr, promontory; jb, jugular bulb; Coch, cochlea; cg, cholesterol granuloma; *, cholesterol granuloma in the CT scan; blue arrows, possible drainage pathways.

Conversely, by assessing the anatomical relationship between the position of the cholesterol granuloma of the petrous apex, the infracochlear/perilabyrinthine cellularity and the sphenoid bone on CT scans, two groups of patients were identified (Figure 2):

- group C (Connected): when, from the CT scan study, we detected a connection between the lesion and the infracochlear/perilabyrinthine or sphenoidal cellularity.
- group D (Disconnected): when the CT scan of the apex showed an anatomical condition of disconnection between the cholesterol granuloma cyst and the infracochlear/perilabyrinthine or sphenoidal cellularity.

### 2.3. Signs and Symptoms

For each patient the presence of signs and symptoms was evaluated, and the pure tone and speech and impedance audiometry was assessed.

### 2.4. Type of Surgery

The surgical approach was used for symptomatic cases or for an aggressive growth of the lesion invading the critical structures. The choice of the surgical approach was based on the location and extension of the PACG, on the temporal bone anatomy, and on hearing and other related symptoms. The radiological anatomical features which guided us to decide the proper surgical corridor were the infralabyrinthine and infracochlear cellularity, the sphenoidal sinus pneumatization, the jugular bulb height and the intrapetrous carotid course.

All types of performed surgical interventions were reported and related to the anatomical and hearing conditions of the patient; in particular, four different types of surgical approach were carried out:

- Drainage and marsupialization of the cyst, when a connection between the pneumatized and cellular spaces of the infracochlear/perilabyrinthine area or of the sphenoid area was created.
- Complete resection of the granuloma, in which the whole cyst and its content was removed.
- Subtotal resection of the lesion, in which the cholesterol granuloma was partially removed, leaving a visible portion of the cyst with its capsule in situ, in the absence of a possible drainage route through the infracochlear/perilabyrinthine or sphenoid cellularity.
- Near-total resection, when an almost complete removal of the lesion was achieved, leaving a small part of the capsule without visible cyst content and in the absence of a possible drainage route through the infracochlear/perilabyrinthine or sphenoid cellularity.

### 2.5. Complications and Follow-Up

All intraoperative and both early and late postoperative complications were taken into account, as well as the presence of any recurrences, detected during the follow-up time.

## 3. Results

Out of about 1700 patients, 21 subjects were selected to undergo surgery for the removal of a symptomatic cholesterol granuloma of the petrous apex at our third level clinic. Two of these patients went missing in the post-surgery follow up; for this reason, we decided to take into account only 19 patients (Table 1). They were 12 male and seven female patients, whose average age was 43 with an average follow-up period of 22 months.

### 3.1. MRI Radiological Evaluation

All the magnetic resonance studies showed the typical hyperintense signal both on T1-weighted sequences with or without contrast enhancement and on T2-weighted and flair images, to confirm the presence of cholesterol granuloma.

### 3.2. CT Scan Imaging Study of the Petrous Apex

Concerning the distribution of our patients in the cholesterol granuloma CT scan classification, one out of 19 patients presented a Type A cholesterol granuloma, showing a preserved cellularity of the apex without erosive signs; four out of 19 patients presented a Type B cholesterol granuloma, with erosion and confluence of the apex cellularity without involvement of the bone limitations that cover the noble structures; 14 in 19 patients presented a Type C condition with erosive involvement of the bone limitations of the noble structures of the petrous apex (otic capsule, IAC, dura of the middle and posterior cranial fossa, ICA, jugular bulb).

Concerning the anatomical relation between the PACG and the infracochlear/perilabyrinthine and/or sphenoidal cellularity, patients were classified in this way: nine out of 19 patients belonged to group C (Connected), with a connection between the lesion and the infracochlear/perilabyrinthine and/or sphenoidal cellularity, while ten patients belonged to group D (Disconnected) and did not show any connections between the granuloma and the above-mentioned cellularity.

### 3.3. Sign and Symptoms

Nine patients presented multiple symptoms while ten presented only one symptom.

Analyzing the singular symptoms:

- Eight patients first presented a palsy of the 6th cranial nerve and diplopia.
- Four patients presented temporary and/or permanent spasms or paralysis of the facial nerve.
- Four patients had a deep sensorineural hearing impairment.
- One patient presented a mixed hearing impairment with transmissive prevalence.
- Eleven patients complained about a headache.
- One patient came to our department for meningitis associated with complete facial paralysis and deep sensorineural hearing loss due to a dural interruption near the capsule of the cholesterol granuloma and to the spilling of its cystic content into the pontocerebellar angle.

Regarding the audiometry assessment, the situation was as follows:

- Fourteen patients presented with normoacusis, assessed on the pure tone and speech audiometry.
- One patient presented with moderate mixed hearing loss on the same side of the lesion.
- Four patients showed a pattern of profound sensorineural hearing loss on the same side of the lesion.

### 3.4. Type of Surgery

The surgical approach was decided according to the PACG features in anatomical relation to the structures of the infracochlear/perilabyrinthine and sphenoidal cellularity and to the patients’ hearing conditions.

A total removal of the cholesterol granuloma cyst was performed in seven patients; in four out of seven of them, the transotic associated to infratemporal fossa Type B approach was adopted for the total removal of the cholesterol granuloma cyst from the petrous apex, given the presence of severe sensorineural hearing loss before surgery, and in three cases the absence of connection (group D) between the petrous apex and the infracochlear/perilabyrinthine and sphenoidal cellular structures. Only one case belonged to group C, but given the dimensions of the lesion in a pediatric patient, a total removal was opted for. A transcochlear approach was adopted for one of the seven patients, given the pre-operative presence of a complete facial nerve paralysis that was not reversable through medical treatment, in association with the presence of a deep sensorineural hearing impairment. In the last two patients, who did not present a profound sensorineural hearing loss before surgery, the removal of the lesion was possible via an endoscopic assisted middle cranial fossa approach. For all seven patients, a total removal of the cyst was reached.

In five out of 19 patients, a subtotal or near total removal of the capsule of the cholesterol granuloma was performed. Given the absence of connections (group D) between the petrous apex and the related structures and the presence of a normal hearing function, an endoscopic-assisted middle cranial fossa approach was performed in order to reach the cholesterol granuloma capsule and to remove the petrous apex part among the ICA, the IAC, the trigeminal nerve (V3) and the dura of the middle cranial fossa. Once the cholesterol granuloma was removed, an endoscopic check was performed in order to search any residual disease and, in that case, with the aid of a burr these residues were removed, in particular those underneath the horizontal portion of the intrapetrous ICA. Considering the total number of patients, two of them underwent a near total resection of the cyst, while in three cases the resection was subtotal.

For seven out of 19 patients, the cyst was drained through the fenestration of the infracochlear/perilabyrinthine or sphenoidal cellularity. In three out of seven patients with group C CT scan features and a normal hearing function, a fenestration was performed via a trans-sphenoidal approach, while in another three out of seven patients, an infracochlear approach was carried out to drain the collection. In one patient of this latter subgroup, who presented a Type A cholesterol granuloma, the procedure was not completed due to the anatomical lack of patency and to the reduced space between the carotid artery, the cochlea and the jugular bulb. In the last patient of the group of seven, because of the large dimensions and the compartmentalized aspect of the apex lesion, a trans-sphenoidal drainage route was used in combination with a middle cranial fossa approach to obtain a sphenoidal connection in association with the subtotal removal of the cyst.

### 3.5. Complications and Follow-Up

Complications occurred in two out of 19 surgical procedures. One intraoperative complication emerged during a Type B infratemporal fossa approach associated with a transotic one; that is the fissuration of the vertical portion of the intrapetrous ICA, which was repaired during surgery. An angiography performed immediately after surgery showed a small pseudoaneurysm of the artery which did not need any further neuroradiological intervention. Currently, after several years, the patient is in excellent condition.

One patient out of 19, who had undergone a middle cranial fossa approach, showed a grade IV House Brackman post-operative facial palsy on the 3rd post-operative day and headache. A brain CT scan revealed a subdural hematoma which required surgical evacuation. Facial palsy completely improved in the following 3 months.

During the follow-up period, 17 out of 19 patients initially showed a regression of the symptoms they had shown before surgery and were subsequently stable. One patient who had undergone a trans-sphenoidal drainage presented a new paralysis of the 6th cranial nerve which required a further trans-sphenoidal drainage procedure to close the previously created one through the same surgical route. In one patient out of 19 patients, the infracochlear procedure was interrupted and he is currently being followed-up and presents a stable condition with a Type A petrous apex granuloma without any further development.

## 4. Discussion

Cholesterol granuloma is a benign inflammatory lesion that starts as a tissue reaction to blood degradation products and appears as a round or oval expansive cyst [1].

It produces a smooth osseous expansion shown in the CT scan, as opposed to the irregular erosion which is typical of other lesions such as a metastatic process, paragangliomas, chondromas and cholesteatomas. CT scans show its aggressiveness on the petrous apex bone, its size and extension to the clivus and the anatomical relationship of the lesion with the adjacent anatomical structures. In addition, a temporal bone CT scan becomes imperative for surgery, providing information about the temporal bone pneumatization and guiding in the choice of the surgical access to the petrous apex. In fact, the correlation between the infracochlear, infralabyrinthine and sphenoidal air cells and the PACG can be easily visualized through a CT scan. Also, the height of the jugular bulb is evaluated before surgery. In fact, a high jugular bulb can hinder the access to this petrous apex lesion. Moreover, an aberrant petrous carotid artery can prevent the visibility of the infracochlear tract, making the approach of the same name unsuitable.

MRI images are much more reliable for the diagnosis. This lesion displays high signal intensity on both T1-weighted and T2-weighted images. The increased intensity on T1-weighted images may be due to the combination of cholesterol crystals, chronic hemorrhage and the proteinaceous crystals of which the granuloma is made. Nevertheless, in some cases, it can be difficult to differentiate normal marrow fat from a small cholesterol granuloma, owing to the typically increased signal intensity on T1-weighted images. However, they appear different in the T2-weighted images. In fact, marrow fat exhibits a decreased signal intensity with the T2-weighted (fat saturation) images, while cholesterol granuloma maintains its high signal intensity. MRI scans are mandatory in the preoperative examination of the patient, and they represent the first choice for follow-up after surgery.

In case of involvement or erosion of the carotid artery canal, defined by some authors either as the circumferential involvement of the canal (of at least 180 degrees on the part of the PACG) or as the stenosis of the ICA or as an anterolateral displacement, a preoperative study of the ICA itself through a simple angiography, a CT-angiography or an MRI-angiography is paramount [10].

A differential diagnosis must be made between a cholesterol granuloma and lesions such as a mucocele, a cholesteatoma, a cephaloceles, a petrous apicitis, a petrous apex schwannoma, a chordoma and a chondrosarcoma [11,12].

Patients affected by a PACG may remain asymptomatic for long periods and the lesion may be discovered by chance during a radiological exam. In fact, there is no correlation between its radiological features and its clinical presentation. The outbreak of symptoms is usually related to the expansion of the lesion and its effects on the surrounding structures. From a meta-analysis written in 2019, the most common symptom is hearing loss, followed by headache, vertigo, facial palsy, facial pain or spasms [7]. Sanna and colleagues [8] noted that hearing loss and vertigo are present in approximately 50% of their patients. The compression or irritation of the cisternal segment or the trigeminal ganglion on the ipsilateral side causes facial pain. Diplopia, on the other hand, is related to the compression of the lesion on the sixth nerve in the Dorello canal. Facial spasms result from the irritation of the facial nerve inside the IAC, at the level of the cerebellopontine angle, or inside the fallopian canal. Sensorineural hearing loss, tinnitus and dizziness originate from compression of the eighth nerve or from the erosion of the otic capsule [4,9].

Late signs and symptoms are middle ear effusion, which appears when the eustachian tube is eroded by the granuloma, speech and swallowing problems and seizures, which are very rare [9].

According to Vinciguerra et al. [20], symptoms associated with cholesterol granuloma are mainly related to peri-lesion inflammation rather than its size.

There are not yet universally accepted guidelines in the literature about the management of this lesion in the petrous apex. Two different strategies are proposed to manage it: a “wait and scan” approach and a surgical treatment [20].

### 4.1. Wait and Scan

The “wait and scan” strategy is recommended to patients with a Type A or B PACG, according to our classification, without symptoms, who represent the greatest part, or to symptomatic ones in poor general conditions. Sweeney et al. [13] stated that around 86% of the patients in the follow-up stage showed no radiological progression while only 47.8% of their surgical patients showed an improvement in their symptoms.

The “wait and scan” choice is possible thanks to the benign and slowly growing nature of the lesion. Spontaneous resolutions are also described either due to the spontaneous drainage of the lesion or to the extinction of the inflammatory process propagating it [14].

Conservatively managed patients undergo regular radiological MRI scrutiny to check any possible changes in the lesion dimension or its involvement of petrous apex critical neuro-vascular structures, along with a clinical evaluation to examine the appearance of symptoms. If symptoms show up, the management switches to the surgical treatment [8,13,15].

### 4.2. Surgical Treatment

A surgical approach is usually reserved for symptomatic cases, even if the expansion of the lesion toward critical structures has been described as a possible indication for surgery as well. There are no guidelines indicating which symptoms directly lead us to start a surgical treatment or the proper timing. Drainage or complete removal is indicated in the presence of a symptomatic Type B granuloma or a Type C showing a large extension.

As previously stated, symptoms can be very different, ranging from dizziness and tinnitus to cranial nerve deficits. Also, headaches are sometimes non-specific symptoms, although other possible causes must be investigated. Surgery is indicated only with intolerable headache, despite medical treatment, and when the headache is directly connected to the mass effect of the cholesterol granuloma (e.g., lateralized pain on the same side of the lesion or retro-orbital pain). Therefore, it is necessary to carefully weigh the pros and cons of surgery in this delicate and anatomically complex area.

The choice of which approach to prefer is based on the preoperative hearing status, the location and extension of the lesion, its relationship with neurovascular structures and possible anatomical variations that must be studied through CT and MRI scans before surgery [16,17]. The radio-anatomical features to be evaluated are infralabyrinthine and infracochlear cellularity, sphenoidal sinus pneumatization, jugular bulb height and intrapetrous carotid course.

The aim of surgery is the resolution symptoms and the avoidance of complications related to the state of erosion (facial paralysis, hearing loss, meningitis). In any case, surgery on petrous apex lesions is anatomically challenging due to the nearby presence of critical structures such as cranial nerves, IAC, jugular foramen, inferior petrosal sinus and ICA.

Two surgical options are possible: surgical drainage or a complete surgical removal. However, in some cases, when the anatomy is unfavorable to drainage and the patient has a normal pre-operative hearing function, the removal of the cholesterol granuloma may be partial in order to preserve the hearing function.

#### 4.2.1. Surgical Drainage

The surgical drainage of a cholesterol granuloma is useful for all those patients with normal hearing function and favorable anatomical conditions. It aims at the creation of an opening for the drainage of the cholesterol granuloma in a pneumatic space of the temporal bone and the sphenoid sinus to perform the marsupialization of the granuloma, creating an open and well-ventilated cavity.

This technique entails a minor decompression of the surrounding structures, if compared to the complete removal, and while it is less successful for multiloculated cysts, it shows a lower comorbidity rate. Sanna et al. [8] claim that drainage is preferable to more aggressive resection procedures in patients with PACGs requiring surgery.

The most used surgical drainage approaches are the infracochlear, the infralabyrinthine and the trans-sphenoidal one.

The infracochlear approach follows the air cell tracts below the labyrinth. A bony dissection is carried out in an anterior-medial direction, using the carotid artery as the anterior limit, the round window as the superior limit and the jugular bulb as the posterior limit. This provides an exposure of the antero-inferior and postero-inferior petrous apex. In our experience, the infracochlear route did not provide access to the superior petrous apex because the cochlea blocks the superior extension of the dissection in all cases. If compared to the infralabyrinthine approach, the infracochlear one provides a more direct, shorter, but surely narrower route to reach the apex, and it is contraindicated in high jugular bulb patients. Moreover, it provides less decompression, particularly when draining multiloculated cysts with a thicker content [21].

Traditionally, the infracochlear approach is performed under microscopic view and it requires a postauricular incision. However, another solution is possible, namely a transcanal endoscopic approach. In this case, the postauricular incision is not needed, but the surgeon can still have an excellent visualization of the petrous apex lesion, facilitating the cyst drainage. One disadvantage, however, is represented by the one-handed nature of the technique [22]. Mattox claims that the endoscope can be useful to verify the complete drainage and for the identification of septa or other divisions [23].

Even if the infracochlear is a hearing-preserving approach, it is possible to have hearing complications such as the impairment of the cochlear arterial or venous vasculature. In fact, the vein of the cochlear aqueduct that drains from the cochlea is located in the surgical corridor. Sanna et al. reported 2.9–25% of hearing loss cases after an infracochlear or an infralabyrinthine approach [8,24].

A careful study of the anatomy on CT scans is crucial to understand whether the infracochlear cellularity and the space between the vertical ICA—the gulf of the jugular vein and the promontory—is sufficient to allow for an adequate drainage and ventilation of the cholesterol granuloma petrous apex cavity. In our case series, we had to interrupt the infracochlear approach, selected for a single patient, due to the insufficient width of the surgical window to the petrous apex we had managed to create. In this case, the PACG was reached, but this may not be sufficient to grant a stable result since the possible restenosis of the created connection is inversely proportional to the width of this anatomical window.

The infralabyrinthine approach allows for a wider exposure of the petrous apex if compared to the infracochlear one. It requires a transmastoid bone removal between the semicircular canals, the jugular bulb and the mastoid portion of the facial nerve. A high jugular bulb can hinder the dissection even when a wide mastoidectomy is carried out. This type of approach is indicated when there is a good pneumatization of the infralabyrinthine cells [15].

The trans-sphenoidal approach is indicated for lesions that contact or invade the sphenoid sinus and that are accessible via the posterior wall of the sphenoid, as well as for patients with a large, well-pneumatized sphenoid sinus. However, this third surgical drainage pathway is not achievable when the ICA runs between the cholesterol granuloma and the sphenoid sinus.

**Figure 3:**
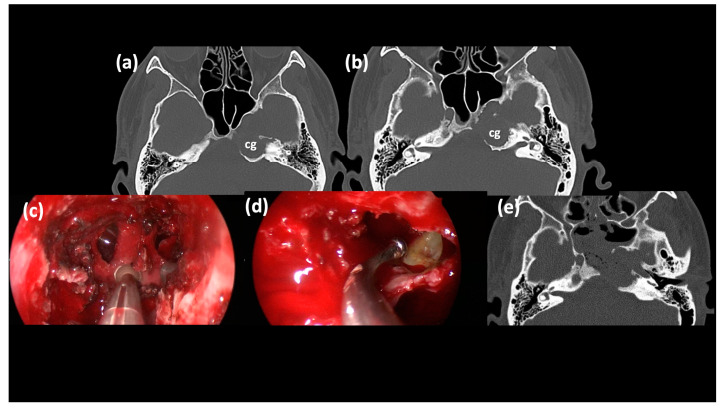
Trans-sphenoidal approach. (**a**,**b**) Axial CT scan sections of a left PACG. (**c**) Surgical step: with a diamond bur the sphenoid sinus is opened. (**d**) Surgical step: with a curve dissection tool the cyst of the PACG is opened and drained. (**e**) Post-surgery CT scan with regular trans-sphenoidal drainage. cg, cholesterol granuloma.

This latter technique is minimally invasive, its rate of post-operative facial weakness is very low and the surgical and hospitalization times are shorter, if compared to the ones of the infralabyrinthine and infracochlear approaches. Additionally, the endoscopic visualization allows for the magnification of important structures adjacent to the sphenoid sinus and grants a wide marsupialization of the cholesterol granuloma (Figure 3) [25].

Finally, a nasoseptal flap is a good option to prevent epithelial growth and stenosis of the created opening after marsupialization, and in some cases a silastic tube can be added to furtherly grant patency [26]. In a systematic review, the stent placement appears to prevent recurrence in a higher number of cases. In fact, Eytan at al. [27] reported a recurrence rate difference between the use (10.7%) and the omission (4.3%) of a postoperative stenting; however, this difference was a non-statistically significant one and it must be considered that restenosis can be asymptomatic and may be conservatively managed.

#### 4.2.2. Complete Surgical Removal

The complete surgical resection entails the removal of the cyst walls and content, and its indications are symptom recurrence after drainage or the treatment of patients who are surgical candidates presenting a pre-operative severe-profound sensorineural hearing loss on the side of the PACG.

The radical approaches are the Type B infratemporal fossa approach in combination with a transotic one and the middle cranial fossa approach. These surgical routes provide excellent control of the lesion and allow for a complete removal. In fact, the extensive granuloma pseudo-capsule resection with the obliteration of the cavity appears to avoid the re-accumulation of cyst content and can potentially grant a complete resolution of the pathology, as opposed to the mere surgical drainage. A radical removal is, however, technically more challenging than a drainage procedure and it has a higher complication rate [17,18].

**Figure 4:**
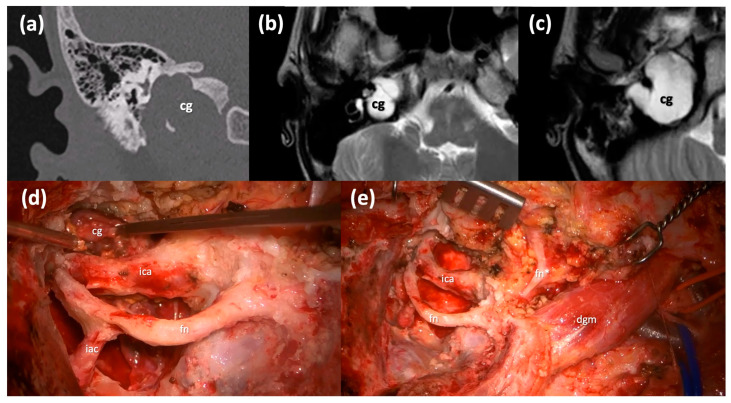
Type B infratemporal fossa approach combined with a transotic approach. (**a**) Coronal CT scan of a massive right Type C PACG. (**b**,**c**) Axial T2-weighted MRI sections of the same PACG showing its hyperintense signal and its relationship with the IAC, the ICA and the clivus. (**d**) Surgical step: the facial nerve has been completely skeletonized from the IAC to the stylomastoid foramen and it is left in a bridge-like fashion over the surgical field; careful maneuvers are employed to dethatch the PACG from the horizontal segment of the ICA. (**e**) Surgical step: final surgical field where the PACG has been completely removed. cg, cholesterol granuloma; ica, internal carotid artery; fn, mastoid segment of the facial nerve; iac, internal auditory canal; fn*, intraparotid facial nerve; dgm, digastric muscle.

The Type B infratemporal fossa route in combination with a transotic one is selected in case of a pre-operative severe-profound sensorineural hearing impairment since it sacrifices the hearing function, and it requires the blind closure of the external auditory skin. This technique is based on the skeletonization of the facial nerve, which is kept suspended in its bone canal from the stylomastoid foramen to the fundus of IAC, and on the skeletonization of the intrapetrous ICA in its vertical and horizontal portions. This is possible through the anterior transposition of the mandibular condyle and the middle meningeal artery and the V3 trigeminal branch section, to allow for the control of the horizontal portion of the intrapetrous ICA. This approach allows for the removal of the lesion with its capsule from the petrous apex to the clivus anterior to the ICA (Figure 4), while allowing for a direct visualization and a complete control of the whole intrapetrous ICA. Once the cholesterol granuloma has been removed, the surgical cavity is filled with abdominal fat.

This route is suggested if the cholesterol granuloma has a significant extension, and it is closely related to the intrapetrous ICA. In all the cases of intrapetrous ICA involvement, the surgeon must take into account the necessity for a detailed radiological preoperative study of the vessels [8].

An arteriography and a balloon occlusion test of the ICA are crucial in the preoperative study, since an intra-operative injury of the vessel may require its direct closure, thus assessing the presence of a collateral blood circulation is fundamental for the preoperative planning. In our series, we had one case of intrapetrous ICA vertical tract fissuration that we repaired during surgery, and the immediately carried out angiography highlighted a stable pseudoaneurysm that did not require any neuroradiological procedure.

**Figure 5:**
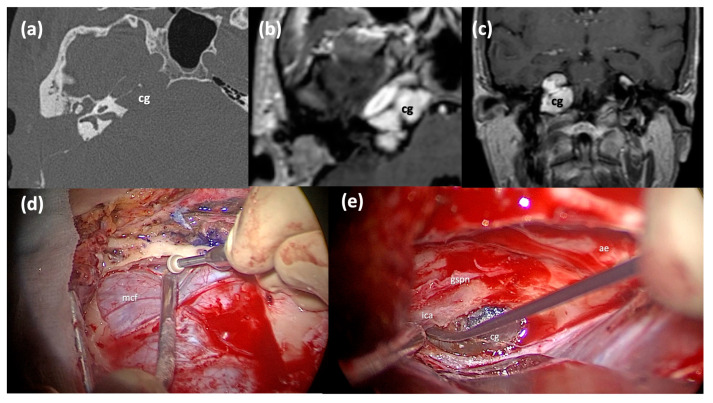
Middle cranial fossa approach. (**a**) Axial CT scan of a right Type C PACG with erosion of the horizontal ICA bone walls. (**b**,**c**) Axial and Coronal T1-weighted MRI sections of the same PACG, showing the typical hyperintense signal. (**d**) Surgical step: a craniotomy has been performed and the dura of the middle cranial fossa is carefully elevated from the skull base. (**e**) Surgical step: the lesion is identified after the Kawase triangle drilling. cg: cholesterol granuloma; mcf, middle fossa dura; gspn, greater superficial petrosal nerve; ae, arcuate eminence; ica, internal carotid artery.

On the other hand, for what concerns the middle cranial fossa approach, this is chosen for patients for whom a fenestration is not possible due to unfavorable anatomical conditions, with a preoperative normal hearing function or a serviceable hearing. It is usually proposed for cholesterol granulomas that extend into the middle fossa, medial to the ICA [15]. This surgical route often allows the pseudo-capsule to be completely removed; however, it is very difficult to achieve a stable aeration of the cavity, which must be obliterated. In some cases, a drainage route can be created through the tegmen tympani. The middle cranial fossa approach is based on a temporal craniotomy (4 × 4 cm) above the zygomatic process, the subsequent exposure of the middle cranial fossa dura and the elevation of the temporal lobe from the floor of the middle cranial fossa. The greater superficial petrosal nerve and the horizontal tract of the underlying intrapetrous ICA are identified at the level of the petrous apex. In the posterior portion of the field the IAC is identified while anteriorly the middle meningeal artery is coagulated and severed. The mandibular branch of the trigeminal nerve within the foramen ovale is identified as the anterior limit of the approach. Finally, after the elevation of the dura of the middle fossa, the petrous apex bone between the above-mentioned identified structures (Kawase triangle) is drilled to reach the cholesterol granuloma, which is subsequently removed (Figure 5).

In the majority of cases treated with this approach, it is difficult to create a drainage fenestration of the cholesterol granuloma cavity towards the mastoid or tympanic or sphenoidal sinus spaces. Even the total removal of the cysts is not always possible, especially if the granuloma develops well underneath and lateral to the horizontal portion of the ICA. The implementation of the endoscope in combination with the microscopic technique has allowed for the optimization of the removal of the granuloma through this approach. However, in most cases, residues of the pseudo-capsule may remain. Therefore, when a proper drainage corridor is not easily achievable, a middle cranial fossa approach can be used to perform a subtotal resection, leaving part of the cystic content behind, or a near-total resection, leaving only part of the pseudo-capsule in the surgical cavity. In all these cases, a follow-up is necessary over the years for a possible recurrence of the cholesterol granuloma formation or when symptoms reappear.

One of the possible risks, both during surgery and in very rare cases of non-operated patients when a cholesterol granuloma spontaneously evolves, is the creation of a fistula that is a connection between the granuloma content and the cerebellopontine angle, with the possible contamination of the subarachnoid spaces yielding to a chemical meningitis. For one of our patients the diagnosis of cholesterol granuloma was made because the patient presented a chemical meningitis caused by the spontaneous fissuration of the granuloma pseudo-capsule and the opening of the content into the pontocerebellar angle and the dura. This patient presented meningitis, facial nerve palsy and a profound sensorineural hearing loss. A transcochlear approach was therefore scheduled for the total removal of the cholesterol granuloma.

**Figure 6:**
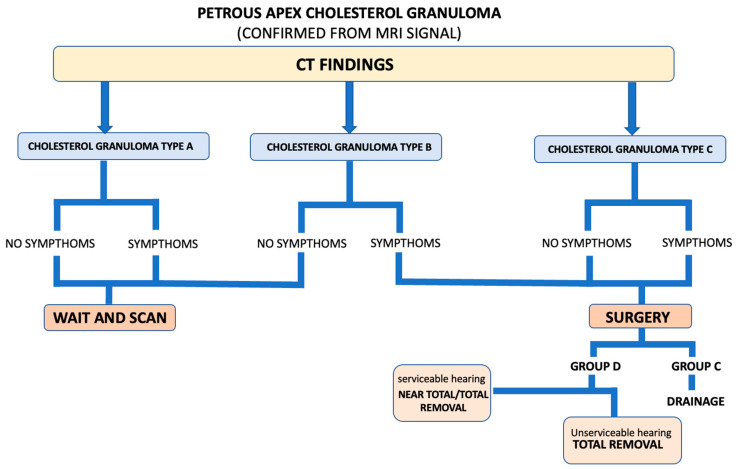
Decision-making flow chart based on our CT classification of the PACG.

From our study based on the radiological appearance of the petrous apex on the CT scan, we classified three types of cholesterol granuloma (A, B and C) based on the remodeling of the petrous apex bone caused by the lesion. This radiological classification of PACGs could be helpful in the decision-making flowchart of the patient suffering from this lesion (Figure 6).

In most cases displaying the typical features of a cholesterol granuloma, the CT scan showed well-preserved cells without confluence or bone erosion. In these cases, it is possible to hypothesize an initial stage without the presence of a pseudo-cystic appearance of the lesion, which necessarily implies a remodeling of the petrous apex bone. Thus, these cases were classified as Type A, from a radiological point of view (CT scan), and they showed a petrous apex with regular bone cells without erosion and confluence. From our case series, only one symptomatic patient presented this type of cholesterol granuloma, and he experienced conductive hearing loss and headache, while all the other symptomatic patients in this series showed Type B or C lesions. Type A cholesterol granuloma is probably an initial asymptomatic stage and, when symptoms are present, their actual correlation with the radiological presence of a cholesterol granuloma must be carefully assessed and a “wait and scan” strategy is a reasonable management. In fact, the only patient with a Type A lesion in our case series underwent an infracochlear approach, which was then suspended due to his unfavorable anatomy, and currently presents no lesion evolution.

The management could be different in patients suffering from Type B cholesterol granuloma. In case of bone erosion and confluence of the apex cells visible on the CT scan, it is necessary to consider the patient’s symptoms. In the opinion of the authors, asymptomatic patients can be followed through a “wait and scan” protocol and their evolution can be assessed over time, while symptomatic patients should be surgically treated.

For patients with a Type C PACG, already presenting an erosion of the petrous apex involving the bone limitations of the critical structures in this area, the management must necessarily be surgical, even in the absence of symptoms, to avoid likely complications in case of lesion evolution, such as the previously mentioned chemical meningitis due to cholesterol granuloma fistula one of our patients developed.

In addition, relying on CT scans, for each type of PACG, we decided to divide the three types (A, B, C) into two subgroups (C—connected and D—non connected) based on the presence or lack of connection of the PACG with the infracochlear/perilabyrinthine and sphenoidal cellularity. This subdivision can inform us on the presence of favorable anatomical conditions (group C) to perform a fenestration/drainage of the cholesterol granuloma through the infralabyrinthine, infracochlear or trans-sphenoidal corridors. In contrast, in case of a lack of connection to the air cell tracts (group D), we can opt for a total/subtotal/near-total removal (via a middle cranial fossa approach) in case of good hearing or for a total removal (via a combination of a transotic and infratemporal fosse Type B approaches) in case of profound pre-operative sensorineural hearing loss.

It must also be considered that cholesterol granuloma is a recurring pathology, especially in the case of fenestration or subtotal removal and that over time the recurrence of symptoms can be associated to a new growth of the lesion. The follow-up of surgically treated patients is based on the clinical examination, including an audiometric and vestibular evaluation and on imaging. A recurrence can be expected especially in patients undergoing a subtotal removal or an insufficiently effective drainage of the lesion. For these patients, if symptoms reappear, a further surgical intervention should be planned.

However, we must consider that there is no association between the restenosis of a surgically created drainage pathway and the recurrence of symptoms. For this reason, in cases of stenosis, a conservative wait-and-scan approach is proposed, while revision surgery is planned only in case of significant symptoms.

PACG can become a recurrent disease when the patient starts to be symptomatic again, or when the lesion shows a growing behavior on imaging, and revision surgery must be considered for these patients. According to the literature, recurrence happens in about 14–16% of the patients who underwent surgery, while the mere presence of fluid secretion in an aerated cavity is not to be considered a recurrent lesion. The main cause for recurrence is the closure of either the surgically positioned drainage catheter or fenestration or the impossibility of achieving a good ventilation of the cavity during surgery. At the same time, there is no evidence of a correlation between any pseudo-capsule residue on the surgical cavity and the rate of recurrence [8].

## 5. Conclusions

While the management of PACGs is still controversial, according to our classification and surgical outcomes, Type A, being mostly asymptomatic, should be managed with “wait and scan”, Type B should undergo surgery when symptoms are present, while Type C should always undergo surgery because of their invasiveness and potential complications. When a connection is present between the lesion and the infracochlear, perilabyrinthine and sphenoidal air cells, a drainage should be attempted; otherwise, a surgical resection is chosen, and its completeness depends on the preoperative general and hearing status of the patient and the chosen surgical approach.

## Figures and Tables

**Table 1:** Patients’ cohort. MCF, middle cranial fossa; IFTB, infratemporal fossa Type B. approach.

Name	Sex	Age at Surgery	Symptoms	Surgical Approach	Follow Up	Complication
**CM**	F	64	Headache	Trans-sphenoidal	1 month, no symptoms.	
**EA**	M	36	Headache + VI cranial nerve paralysis	MCF approach	6 months, MRI negative, no symptoms	
**FS**	M	48	Headache + VI cranial nerve paralysis	Trans-sphenoidal	Recurrence with several surgeries following years. No symptoms at the end.	
**LLA**	F	46	Headache	MCF approach	6 months, MRI negative, no symptoms	
**SA**	F	15	Recurrent facial paralysis	MCF approach	1 year, MRI negative, no symptoms	
**TG**	M	50	Headache + hearing loss	Transotic + IFTB	3 years, MRI negative, no symptoms	ICA fissuration
**BG**	M	59	VI cranial nerve paralysis	Trans-sphenoidal + MCF approach	1 years, MRI with minimal residual, no symptoms.	
**KWK**	M	40	Hearing loss	Transotic + IFTB	1 month, no symptoms.	
**NPE**	F	57	Headache	Partial removal with infracochlear approach	3 years, MRI with stable residual	
**MM**	M	19	VI cranial nerve paralysis	Partial removal with infracochlear approach	3 years, MRI with stable residual	
**GL**	F	53	VI cranial nerve paralysis	MCF approach	6 years, MRI negative, no symptoms	
**VE**	F	11	Headache	MCF approach	1 year, MRI negative, no symptoms	
**MG**	M	49	Headache + Facial nerve paralysis	MCF approach	3 months, no symptoms	Transient facial palsy + Subdural hematoma
**BI**	F	52	Headache + transmissive hearing loss	Infracochlear	2 years, stable	
**GP**	M	53	VI cranial nerve paralysis + hearing loss	Transotic + IFTB	3 years, MRI negative, no symptoms	
**CF**	F	81	VI cranial nerve paralysis	Trans-sphenoidal	6 months, CT negative, no symptoms	
**AA**	M	35	VI cranial nerve paralysis + headache	MCF approach	1 year, MRI negative, no symptoms	
**NA**	M	35	Facial nerve paralysis + hearing loss + Headache + vertigo + meningitis	Transcochlear	5 years, no symptoms	
**NCK**	M	12	Facial nerve paralysis + hearing loss	Transotic + IFTB	6 months, MRI negative, no symptoms	

## Data Availability

The original contributions presented in the study are included in the article, further inquiries can be directed to the corresponding author.
